# Finite element analysis for ternary hybrid nanoparticles on thermal enhancement in pseudo-plastic liquid through porous stretching sheet

**DOI:** 10.1038/s41598-022-12857-3

**Published:** 2022-06-02

**Authors:** Muhammad Sohail, Essam R. El-Zahar, Abd Allah A. Mousa, Umar Nazir, Saad Althobaiti, Ali Althobaiti, Nehad Ali Shah, Jae Dong Chung

**Affiliations:** 1 Department of Mathematics, Khwaja Fareed University of Engineering & Information Technology, Rahim Yar Khan, 64200 Pakistan; 2 Department of Mathematics, College of Science and Humanities in Al-Kharj, Prince Sattam Bin Abdulaziz University, P.O. Box 83, Al-Kharj, 11942 Saudi Arabia; 3 Department of Basic Engineering Science, Faculty of Engineering, Menoufia University, Shebin El-Kom, 32511 Egypt; 4 Department of Mathematics, College of Science, Taif University, P.O. Box 11099, Taif, 21944 Saudi Arabia; 5 Department of Applied Mathematics and Statistics, Institute of Space Technology, P.O. Box 2750, Islamabad, 44000 Pakistan; 6 Department of Sciences and Technology, Ranyah University Collage, Taif University, P.O. Box 11099, Taif, 21944 Saudi Arabia; 7 Department of Mechanical Engineering, Sejong University, Seoul, 05006 Korea

**Keywords:** Mathematics and computing, Nanoscience and technology

## Abstract

Thermal performance can be enhanced due to the mixing of nanoparticles in base fluid. This research discusses the involvement of ternary hybrid nanoparticles in the mixture of pseudo-plastic fluid model past over a two dimensional porous stretching sheet. Modelling of energy equation is carried out in the presence of external heat source or sink and viscous dissipation. The flow presenting equations and derived in Cartesian coordinate system under usual boundary layer theory in the form of complex coupled partial differential equations (PDEs). The derived PDEs have been converted into corresponding ordinary differential equations (ODEs) with the engagement of suitable transformation. The engineers, scientists and mathematicians have great interest in the solution of differential equations because to understand the real physics of the problem. Here, finite element scheme has been used to approximate the solution of the converted problem. The contribution of several emerging parameters on solution have been displayed through graphs and discussed. It is recommended that the finite element method can be engaged to approximate the solution of nonlinear problems arising in modelling the problem in mathematical physics.

## Introduction

Several important rheological relations exist to predict the nature and rheological behaviour of different materials. Researchers, mathematicians and engineers have the thrust to notice the comportment of different materials by varying the parameters involved in the governing laws. By viewing the internal properties and the practical usage several important relations exist in the literature. Pseudo-plastic material^1–6^ is an important model. Several important works have been reported with different physical effects in different geometries with different techniques. For instance, Hemeida^1^ studied pseudo-plastic behaviour in porous medium and presented the analytic solution for developed modelled problem. Contribution of gravity effects are ignored during his work. Desouky and Al-Awad^2^ developed the model for yield exhibiting pseudo-plastic material which discusses the parametric impacts on laminar and turbulent phases. Zhang et al.^3^ developed a new mathematical model to capture the characteristics of pseudo-plasticity of materials by taking the different magnetic field strength magnitudes. Yoshino et al.^4^ used lattice Boltzmann procedure to studied the rheology of non-Newtonian power law and pseudo-plastic materials. Hina et al.^5^ examined the involvement of flexible wall properties on peristaltic flow of pseudo-plastic model in a curved channel with heat and mass transfer. They developed the mathematical model in curvilinear coordinates and simplified with the assumptions of long wavelength. They presented the series solution using perturbation approach and the impact of numerous involved parameters has been explored by plotting the graphs and tables. Heat transfer in pseudo-plastic nanomaterial flow with viscous dissipation past over a permeable surface by mixing Al_2_O_3_, Cu, CuO_2_ and TiO_2_ in sodium carboxyl-methyl based fluid model was reported by Maleki et al.^6^. They solved the resulting converted ODEs numerically and several plots are shown for numerous involved parameters on velocity and fluid temperature. They analysed the decline in fluid velocity for higher volume fraction involvement and increase in temperature profile. Furthermore, they noticed the augmentation in temperature field for higher estimation of Eckert number.

Fluid face interaction with the involvement of nanoparticles is essential to boost the thermal performance. Researchers have great interest to monitor the thermal performance and stability of the system by mixing the nanoparticles in base fluid. Several important contributions are reported to investigate the involvement of nanoparticles in different fluids. For instance, Ali et al.^7^ presented the generalized fluid model namely Brinkman type material with non-integer order derivative having non-singular kernel to examine the features of shape factors on engine oil base fluid in rotating frame with oscillatory boundary conditions. They engaged Laplace transform procedure to approximate the solution of arising problem with heat transfer. Muhammad et al.^8^ studied the contribution of CNTs in Casson model in rotating frame with MHD effect, heat generation and radiation past over a rotating stretched surface. They engaged boundary layer approximation to reduce the arising PDEs into simplified structure and then converted into ODEs by using appropriate transformation. They computed the solution with well-known homotopic procedure. They established that for CNTs based material velocity field decreases and temperature upsurges against higher estimation of rotation parameter. Mixed convective flow past over a porous stretching cylinder having CNTs mixtures with slip conditions and heat generation was studied by Hayat et al.^9^ via shooting method. They monitored the significant increase in fluid velocity for higher estimation of porosity parameter and magnetic parameter. Ghadikolaei et al.^10^ scrutinized mixed convective magneto Casson nanofluid model with Joule heating past over a stretching inclined porous sheet. They observed the increase in velocity for higher estimation of angle of inclination and it decreases for fluid parameter and magnetic parameter. Aman et al.^11^ studied different nanoparticles mixture with Maxwell model to monitor their contribution to enhance heat transfer. They engaged Laplace transform method to solve the modelled equations. Rashid et al.^12^ computed the exact solution for the involvement of metallic nanoparticles in CNTs based fluid mixture past over a two-dimensional aligned stretching sheet. They reported the increase in temperature field by increasing the amount of volume fraction, magnetic parameter and inclination angle. Some novel contributions covering the modelling of transport problem are listed in Refs.^13–19^. Hosseinzadeh et al.^20^ investigated hydrothermal aspects in non-Newtonian martial involving nanoparticles in porous vessels. Hosseinzadeh et al.^21^ developed model related hybrid nanoparticles approach in porous fin wetted considering trapezoidal in the presence of convex cross and concave parabolic sections. TalebiRostami et al.^22^ used RBF method to simulate consequences of dusty hybrid nanoparticles in porous media under action of magnetic field. Hosseinzadeh et al.^23^ studied the role of magnetic field in entropy generation phenomena in the presence of hybrid nanoparticles towards a stretching surfaces considering by non-linear thermal radiation. Ali et al.^24^ implemented finite element method to simulate numerical and graphical results of developed model based on micro-polar fluid in the presence of magnetic field across an inclined surface. Khan et al.^25^ discussed study of magnetic dipole in stagnation point flow in the occurrence of thermal radiation inserting nanofluid past a vertical surface using FEM. Ali et al.^26^ analysed numerical aspects of Maxwell fluid along with nanoparticles under action of Falkner–Skan flow considering activation energy approach past a wedge. Khan et al.^27^ studied impacts of magneto-hydrodynamic axisymmetric in the presence of nanoparticles over a stretching surface. Ali et al.^28^ captured consequences of non-Newtonian martial along with magnetic field over a heated surface. They implemented FEM to conduct numerical consequences. Ali et al.^29^ adopted finite element method to know numerical results of model containing consequences of magnetic dipole inserting the impact of hybrid nanofluid over a stitching frame. Ali et al.^30^ discussed features of mass diffusion and heat energy in Maxwell liquid considering nanofluid over a stretching surface involving chemical reaction and thermal radiations.

A significant review published predicts that comparative thermal performance among tri-hybrid nanoparticles, nanoparticles and hybrid nanoparticles in pseudo-plastic liquid considering ethylene glycol over heated vertical surface is not performed yet. Heat source and Darcy’s Forchheimer law are implanted. Moreover, develop model is simulated by finite element method. Available exploration does not show the comportment of ternary hybrid nanoparticles mixture in pseudo-plastic model with heat transfer aspects. This novel contribution fills this gap and will provide a foundation to extend this research with other existing fluid models under several important physical considerations. This research is designed with following fashion

**Figure 1:**
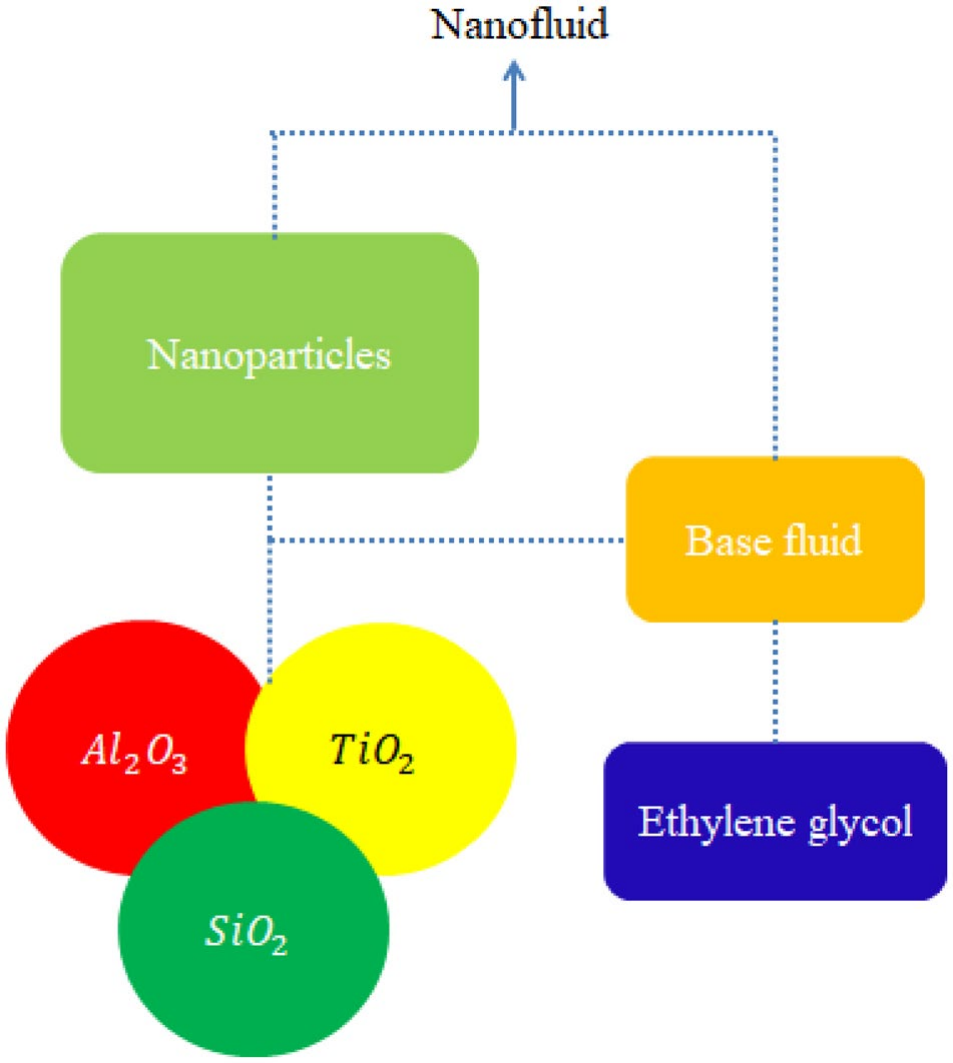
Prepared scheme of tri-hybrid nanoparticles.

- Literature survey is presented in “Introduction”;
- Modelling is presented in “Description of model”;
- “Numerical approach” covers the methodology along with grid independent analysis;
- Comparative study and detailed discussion and description of obtained solution against numerous involved parameters have been reported in “Graphical discussion and outcomes” and important findings are listed in “Key findings”. Prepared scheme of tri-hybrid nanoparticles, nanoparticles and hybrid nanoparticles is considered by Fig. 1.

## Description of model

**Figure 2:**
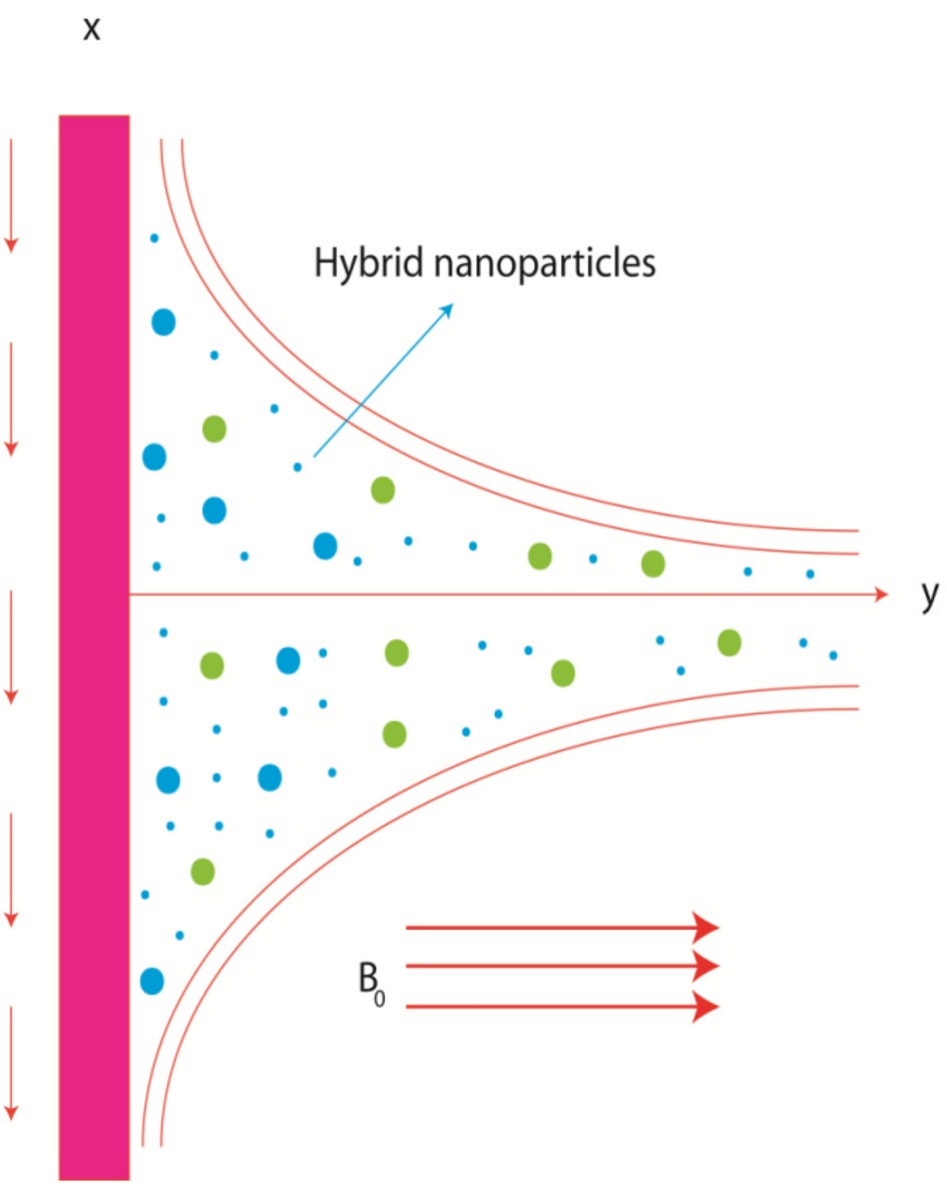
Geometrical view of flow model.

Argumentation of heat transfer in rheology of in pseudo-plastic liquid (non-Newtonian) due to inclusion of tri-hybrid nanomaterials is observed. A heated vertical surface is taken to visualize thermal aspects. Boundary layers are generated because of moving wall along horizontal direction. The phenomenon of heat source and heat observation is addressed in energy equation. The concept of viscous dissipation is also addressed. Correlations among nanofluid, hybrid nanoparticles, tri-hybrid nanoparticles are assumed by Table 1 while schematically view of model is considered by Fig. 2.

**Table 1:** Thermal properties for ethylene glycol, aluminium oxide, titanium dioxide and silicon dioxide in [33 and 34].

Nanoparticles	$K$ (thermal conductivity)	$\sigma$ (electrical conductivity)	$\rho$ (desity)
${\mathrm{C}}_{2}{\mathrm{H}}_{6}{\mathrm{O}}_{2}$	0.253	$4.3\times {10}^{-5}$	1113.5
${\mathrm{Al}}_{2}{\mathrm{O}}_{3}$	32.9	$5.96\times {10}^{7}$	6310
${\mathrm{TiO}}_{2}$	8.953	$2.4\times {10}^{6}$	4250
${\mathrm{SiO}}_{2}$	1.4013	$3.5\times {10}^{6}$	2270

Formulation of PDEs is obtained via (boundary layer approximations) BLAs. PDEs^6,31,32^ are

1 $$\frac{\partial {u}_{1}}{\partial x}+\frac{\partial {u}_{2}}{\partial y}=0,$$

2 $${u}_{1}\frac{\partial {u}_{1}}{\partial x}+{u}_{2}\frac{\partial {u}_{1}}{\partial y}={\nu }_{Thnf}\frac{\partial }{\partial y}\left({\left|\frac{\partial {u}_{1}}{\partial y}\right|}^{m-1}\frac{\partial {u}_{1}}{\partial y}\right)-\frac{{\nu }_{Thnf}}{{k}^{s}}{F}_{D}{u}_{1}-\frac{{F}_{D}}{{\left({k}^{s}\right)}^\frac{1}{2}}{{u}_{1}}^{2}+{G\gamma }_{Thnf}\left(T-{T}_{\infty }\right),$$

3 $${u}_{1}\frac{\partial T}{\partial x}+{u}_{2}\frac{\partial T}{\partial y}=\frac{{k}_{Thnf}}{{(\rho {C}_{p})}_{Thnf}}\frac{{\partial }^{2}T}{{\partial }^{2}y}+\frac{Q\left(T-{T}_{\infty }\right)}{{(\rho {C}_{p})}_{Thnf}}+\frac{{\mu }_{Thnf}}{{(\rho {C}_{p})}_{Thnf}}{\left|\frac{\partial {u}_{1}}{\partial y}\right|}^{m+1}.$$

Theory related to no-slip provides following boundary conditions^31,32^.

$${u}_{1}={U}_{w}, {u}_{2}=-{V}_{w}, T={T}_{w}\mathrm{ at }y=0,$$

4 $${u}_{1}\to {u}_{\infty }, T\to {T}_{\infty }\mathrm{ when }y\to \infty .$$

Variation in variables of model^31–33^ and is

5 $$\theta =\frac{T-{T}_{\infty }}{{T}_{w}-{T}_{\infty }}, \eta ={y\left(\frac{{U}^{2-m}}{x{\nu }_{f}}\right)}^{\frac{1}{m+1}},\Psi =F{\left(x{\nu }_{f}{U}^{2m-1}\right)}^{\frac{1}{m+1}}.$$

Equation (5) is implemented in PDEs (partial differential equations) and formulated ODEs (ordinary differential equations) are

6 $${\left({\left|F{^{\prime}}{^{\prime}}\right|}^{m-1}F{^{\prime}}{^{\prime}}\right)}^{^{\prime}}+\frac{1}{m+1}{F}^{{^{\prime}}{^{\prime}}}F-\epsilon {F}^{^{\prime}}-\frac{{\nu }_{f}}{{\nu }_{Thnf}}{F}_{R}\left({{F}^{^{\prime}}}^{2}\right)+\frac{{\nu }_{f}}{{\nu }_{Thnf}}\lambda \theta =0,$$

7 $${\theta }^{{^{\prime}}{^{\prime}}}+\frac{Pr}{m+1}F{\theta }^{^{\prime}}-\frac{{k}_{f}{\left(\rho {C}_{p}\right)}_{hnf}}{{k}_{hnf}{\left(\rho {C}_{p}\right)}_{f}}PrEc{\left|F{^{\prime}}{^{\prime}}\right|}^{m+1}+\frac{{k}_{f}}{{k}_{hnf}}{H}_{t}Pr\theta =0.$$

Correlations among tri-hybrid nanoparticles, hybrid nanoparticles and nanofluid^31,32^ are

8 $${\rho }_{Thnf}=\left(1-{\varphi }_{1}\right)\left\{\left(1-{\varphi }_{2}\right)\left[\left(1-{\varphi }_{3}\right){\rho }_{f}+{\varphi }_{3}{\rho }_{3}\right]+{\varphi }_{2}{\rho }_{2}\right\}+{\varphi }_{1}{\rho }_{1},$$

9 $$\frac{{\mu }_{f}}{{\left(1-{\varphi }_{3}\right)}^{2.5}{\left(1-{\varphi }_{2}\right)}^{2.5}{\left(1-{\varphi }_{1}\right)}^{2.5}}, \frac{{K}_{hnf}}{{K}_{nf}}=\frac{{K}_{2}+2{K}_{nf}-2{\varphi }_{1}\left({K}_{nf}-{K}_{2}\right)}{{K}_{2}+2{K}_{nf}+{\varphi }_{2}\left({K}_{nf}-{K}_{2}\right)},$$

10 $$\frac{{K}_{Thnf}}{{K}_{hnf}}=\frac{{K}_{1}+2{K}_{hnf}-2{\varphi }_{1}\left({K}_{hnf}-{K}_{1}\right)}{{K}_{1}+2{K}_{hnf}+{\varphi }_{1}\left({K}_{hnf}-{K}_{1}\right)}, \frac{{K}_{nf}}{{K}_{f}}=\frac{{K}_{3}+2{K}_{f}-2{\varphi }_{3}\left({K}_{f}-{K}_{3}\right)}{{K}_{3}+2{K}_{f}+{\varphi }_{3}\left({K}_{f}-{K}_{3}\right)},$$

11 $$\frac{{\sigma }_{Tnf}}{{\sigma }_{hnf}}=\frac{{\sigma }_{1}\left(1+2{\varphi }_{1}\right)-{\varphi }_{hnf}\left(1-2{\varphi }_{1}\right)}{{\sigma }_{1}(1-{\varphi }_{1})+{\sigma }_{hnf}(1+{\varphi }_{1})}, \frac{{\sigma }_{hnf}}{{\sigma }_{nf}}=\frac{{\sigma }_{2}\left(1+2{\varphi }_{2}\right)+{\varphi }_{nf}\left(1-2{\varphi }_{2}\right)}{{\sigma }_{2}(1-{\varphi }_{2})+{\sigma }_{nf}(1+{\varphi }_{2})},$$

12 $$\frac{{\sigma }_{nf}}{{\sigma }_{f}}=\frac{{\sigma }_{3}\left(1+2{\varphi }_{3}\right)+{\varphi }_{f}\left(1-2{\varphi }_{3}\right)}{{\sigma }_{3}(1-{\varphi }_{3})+{\sigma }_{f}(1+{\varphi }_{3})}.$$

The parameters are defined as

$$Ec=\frac{{\left({U}_{w}\right)}^{2}}{\left({T}_{w}-{T}_{\infty }\right){\left({C}_{p}\right)}_{f}}, Pr=\frac{U{\left(\rho {C}_{p}\right)}_{f}}{x{K}_{f}}{\left(\frac{{U}^{2-m}}{{\nu }_{f}x}\right)}^{\frac{-2}{m+1}}, {H}_{t}=\frac{{Q}_{0}x}{{\left(\rho {C}_{p}\right)}_{f}U},$$

$$\lambda =\frac{G{\gamma }_{f}x\left({T}_{w}-{T}_{\infty }\right)}{{U}^{2}}, \epsilon =\frac{{{\nu }_{f}F}_{D}}{U}, {F}_{R}=\frac{{F}_{D}x}{\sqrt{{k}^{*}}}.$$

Skin friction coefficient is defined as

13 $${C}_{f}=\frac{2\left({\tau }_{w}\right)}{{\left({U}_{w}\right)}^{2}{\rho }_{f}},{\tau }_{w}={\mu }_{Thnf}{\left(\frac{\partial {u}_{1}}{\partial y}{\left|\frac{\partial {u}_{1}}{\partial y}\right|}^{m-1}\right)}_{y=0}.$$

Simplified form of Eq. (13) becomes

14 $${{\left(Re\right)}^{\frac{1}{m+1}}C}_{f}=-\frac{{\left(1-{\varphi }_{2}\right)}^{-2.5}}{{{\left(1-{\varphi }_{1}\right)}^{2.5}\left(1-{\varphi }_{3}\right)}^{2.5}}\left[{F}^{{^{\prime}}{^{\prime}}}\left(0\right){\left|{F}^{{^{\prime}}{^{\prime}}}\left(0\right)\right|}^{m-1}\right].$$

Nusselt number is dimensionless number which ratio of convective and conductive thermal energy transfer. Nusselt number in the presence of tri-hybrid nano-structures is expressed as

15 $$Nu=\frac{{xQ}_{w}}{{\left({T}_{w}-{T}_{\infty }\right)K}_{f}}, {Q}_{w}=-{K}_{Thnf}{\left.\frac{\partial T}{\partial y}\right|}_{y=0}.$$

Using value of ${Q}_{w}$ and Eq. (16) becomes

16 $${\left(Re\right)}^{\frac{-1}{m+1}}Nu=-\frac{{K}_{Thnf}}{{k}_{f}}{\theta }^{^{\prime}}\left(0\right),$$

where $Re\left(=\frac{{x}^{n}{U}^{2-m}}{{\nu }_{f}}\right)$ is Reynolds number.

### Numerical approach

Finite element algorithm (FEA)^16,24–26^ is addressed to find solution of model. The description of (FEA) is discussed below. Finite element method is most useful approach to handle complex geometries related problems. Most significant role of FEM is that it discretized problem domain number of elements. A finite element method is observed as good method in view of accuracy analysis, convergence analysis and stability analysis rather than others numerical methods.

- Residuals are formulated of Eqs. (6) and (7) within boundary conditions. The obtained residuals of developed problem are
  17 $${\int }_{{\eta }_{e}}^{{\eta }_{e+1}}{w}_{1}\left({F}^{^{\prime}}-H\right)d\eta =0,$$
  18 $${\int }_{{\eta }_{e}}^{{\eta }_{e+1}}{w}_{2}\left[\begin{array}{c}{\left({\left|H{^{\prime}}\right|}^{m-1}H{^{\prime}}\right)}^{^{\prime}}+\frac{1}{m+1}H{^{\prime}}F-\epsilon H-\frac{{\nu }_{f}}{{\nu }_{Thnf}}{F}_{R}\left({H}^{2}\right)\\ +\frac{{\nu }_{f}}{{\nu }_{Thnf}}\lambda \theta \end{array}\right]d\eta =0,$$
  19 $${\int }_{{\eta }_{e}}^{{\eta }_{e+1}}{w}_{3}\left[\theta {^{\prime}}{^{\prime}}+\frac{Pr}{m+1}F\theta -\frac{{k}_{f}{\left(\rho {C}_{p}\right)}_{hnf}}{{k}_{hnf}{\left(\rho {C}_{p}\right)}_{f}}PrEc{\left|H{^{\prime}}\right|}^{m+1}+\frac{{k}_{f}}{{k}_{hnf}}{H}_{t}Pr\theta \right]d\eta =0,$$
- Multiplication of weighted functions within residuals and integrated it over 330 elements. The shape functions of present problem are defined as
  20 $${\psi }_{j}={\left(-1\right)}^{j-1}\left(\frac{-\eta +{\eta }_{j-1}}{-{\eta }_{j}+{\eta }_{j+1}}\right), i=1, 2.$$
- Equations (6) and (7) are termed as strong form while conversion of strong form into weak forms via Galerkin approximation scheme.
- Stiffness elements are derived and these obtained stiffness elements are imposed in assembly approach. Picard linearization scheme is useful to develop system of algebraic equations and simulated iteratively. The stiffness elements are obtained as
  21 $${{K}_{ij}^{11}=0,K}_{ij}^{11}={\int }_{{\eta }_{e}}^{{\eta }_{e+1}}\left(\frac{d{\psi }_{j}}{d\eta }{\psi }_{i}\right)d\eta , {K}_{ij}^{12}=-{\int }_{{\eta }_{e}}^{{\eta }_{e+1}}\left({\psi }_{j}{\psi }_{i}\right)d\eta , {B}_{i}^{1}=0,$$
  22 $${{K}_{ij}^{31}=0,K}_{ij}^{33}={\int }_{{\eta }_{e}}^{{\eta }_{e+1}}\left[-\frac{d{\psi }_{j}}{d\eta }\frac{d{\psi }_{i}}{d\eta }+\frac{Pr}{m+1}\overline{F}\frac{d{\psi }_{j}}{d\eta }{\psi }_{i}+\frac{{k}_{f}}{{k}_{hnf}}{H}_{t}Pr{\psi }_{i}{\psi }_{j}\right]d\eta ,$$
  23 $${K}_{ij}^{32}={\int }_{{\eta }_{e}}^{{\eta }_{e+1}}\left[-\frac{{k}_{f}{\left(\rho {C}_{p}\right)}_{hnf}}{{k}_{hnf}{\left(\rho {C}_{p}\right)}_{f}}PrEc{\left|{H}^{^{\prime}}\right|}^{m}\frac{d{\psi }_{j}}{d\eta }{\psi }_{i}\right]d\eta , {B}_{i}^{2}=0,$$
  24 $${K}_{ij}^{32}={\int }_{{\eta }_{e}}^{{\eta }_{e+1}}\left[-\frac{{k}_{f}{\left(\rho {C}_{p}\right)}_{hnf}}{{k}_{hnf}{\left(\rho {C}_{p}\right)}_{f}}PrEc{\left|{H}^{^{\prime}}\right|}^{m}\frac{d{\psi }_{j}}{d\eta }{\psi }_{i}\right]d\eta , {B}_{i}^{3}=0,$$
  25 $${K}_{ij}^{22}={\int }_{{\eta }_{e}}^{{\eta }_{e+1}}\left[\begin{array}{c}-\left(\overline{{H }^{^{\prime}}}+\left(m-1\right){\left(\overline{{H }^{^{\prime}}}\right)}^{m-2}\right)\frac{d{\psi }_{j}}{d\eta }\frac{d{\psi }_{j}}{d\eta }+\frac{1}{m+1}\overline{F}\frac{d{\psi }_{j}}{d\eta }{\psi }_{i}\\ -\epsilon {\psi }_{i}{\psi }_{j}-\frac{{\nu }_{f}}{{\nu }_{Thnf}}{F}_{R}\left(\overline{H }\right){\psi }_{i}{\psi }_{j}\end{array}\right]d\eta ,$$
  26 $${K}_{ij}^{23}={\int }_{{\eta }_{e}}^{{\eta }_{e+1}}\left[\frac{{\nu }_{f}}{{\nu }_{Thnf}}\lambda {\psi }_{i}{\psi }_{j}\right]d\eta ,{K}_{ij}^{21}=0 .$$
- Convergence is tested via 300 elements and mesh free procedure is made. The stopping criteria is defined as
  27 $$\left|\frac{{\delta }_{i+1}-{\delta }_{i}}{{\delta }^{i}}\right|<{10}^{-5}.$$
- Mesh-free investigation is simulated in Table 2. Table 2 is tabulated to test convergence analysis for 330 elements. The outputs regarding velocity and temperature are recorded at mid of 30–330 elements. It is estimated that results are not affected after 330 elements. Table 3 predicts validation of present problem with already published results by Maleki et al.^6^. Computations are done observing by 330 elements. With the help of Table 3, dimensionless shear stress is compared for the limiting case of performed analysis. From the performed analysis, it is recorded that the obtained results are in well settlement with the findings mentioned in Ref.^6^.

**Table 2:** Illustration of numerical values of $\theta \left(\frac{{\eta }_{Max}}{2}\right)$ and $F{^{\prime}}\left(\frac{{\eta }_{Max}}{2}\right)$ within 330 elements.

Elements	$F{^{\prime}}\left(\frac{{\eta }_{Max}}{2}\right)$	$\theta \left(\frac{{\eta }_{Max}}{2}\right)$
30	0.059668223	0.067321344
60	0.052621388	0.053600561
90	0.050436000	0.049611093
120	0.049374316	0.047715502
150	0.048743940	0.046612898
180	0.048328516	0.045888606
210	0.048036051	0.045375348
240	0.047817660	0.044993943
270	0.047641974	0.044705003
300	0.047504933	0.044472856
330	0.057145238	0.055316303

**Table 3:** Validation of skin friction coefficient with published work for $Pr=0.7, Ec=0, {\varphi }_{1}=0, {\varphi }_{2}=0, {\varphi }_{2}$.

Maleki et al.^6^ ${{-\left(Re\right)}^{\frac{1}{m+1}}C}_{f}$	Present work ${{-\left(Re\right)}^{\frac{1}{m+1}}C}_{f}$
0.44375	0.044705003
0.44375	0.047504933
0.4437	0.048328516

## Graphical discussion and outcomes

Features of tri-hybrid nanoparticles in pseudo-plastic liquid are noticed. Heat generation and viscous dissipation is addressed. Law of Darcy’s Forchheimer is taken out into flow of pseudo-plastic liquid. Finite element scheme is utilized to capture results of current investigation. Study of various parameters on flow and thermal energy is tabulated in view of tables and graphs.

### Aspects of physical parameters on velocity curves

**Figure 3:**
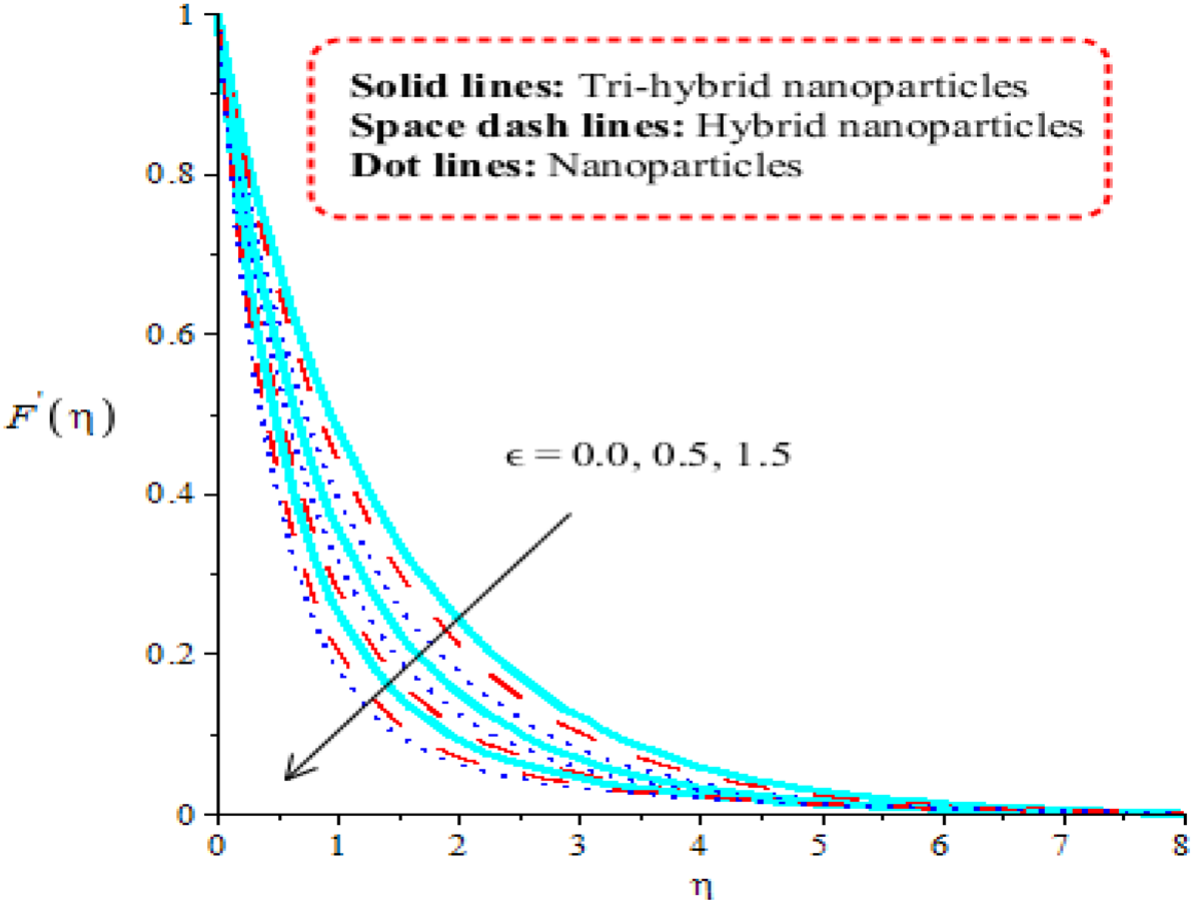
Distribution in ${F}^{^{\prime}}(\eta )$ against $\epsilon .$

**Figure 4:**
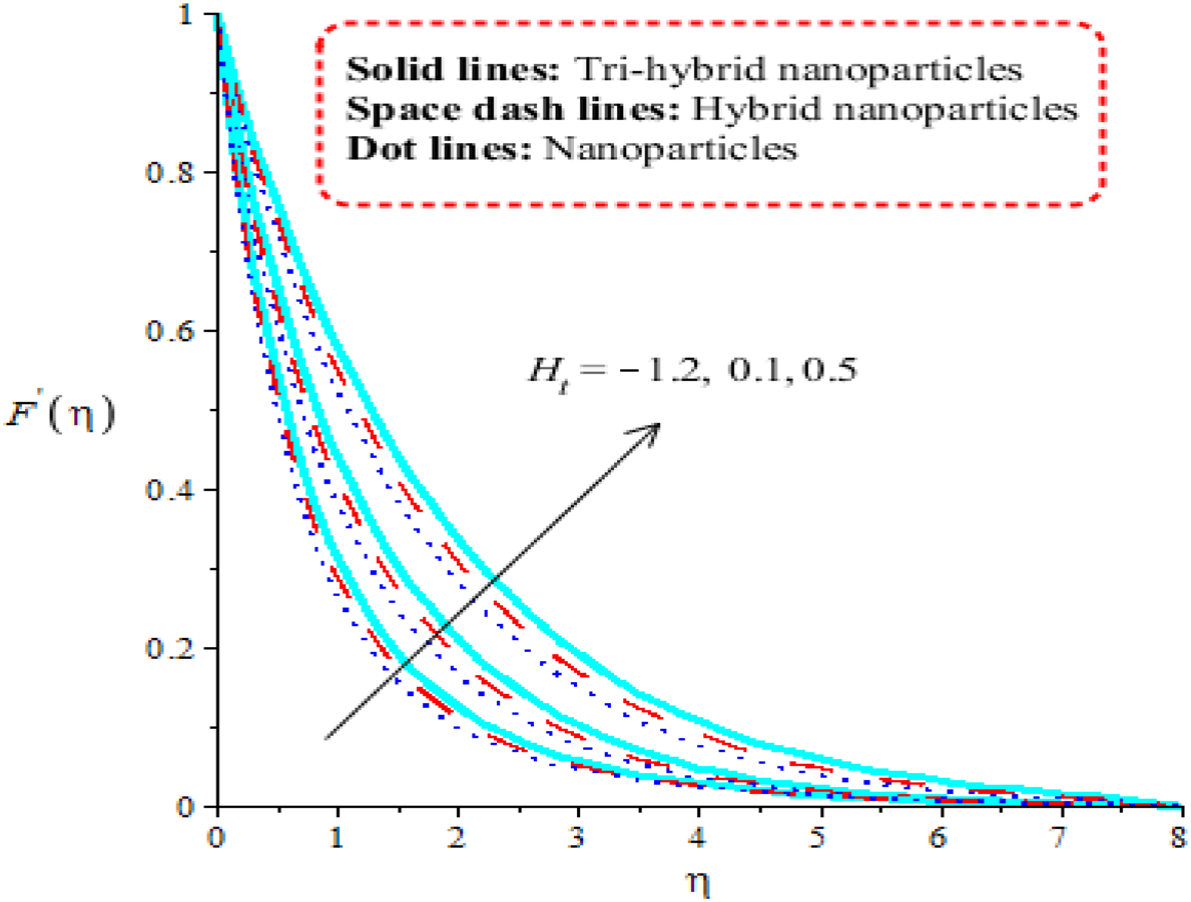
Distribution in ${F}^{^{\prime}}(\eta )$ against ${H}_{t}.$

**Figure 5:**
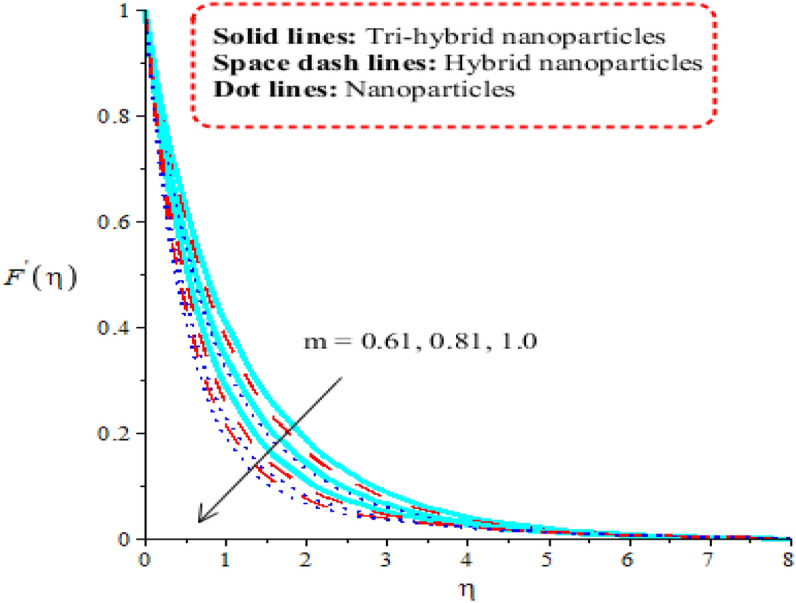
Distribution in ${F}^{^{\prime}}(\eta )$ against $m.$

**Figure 6:**
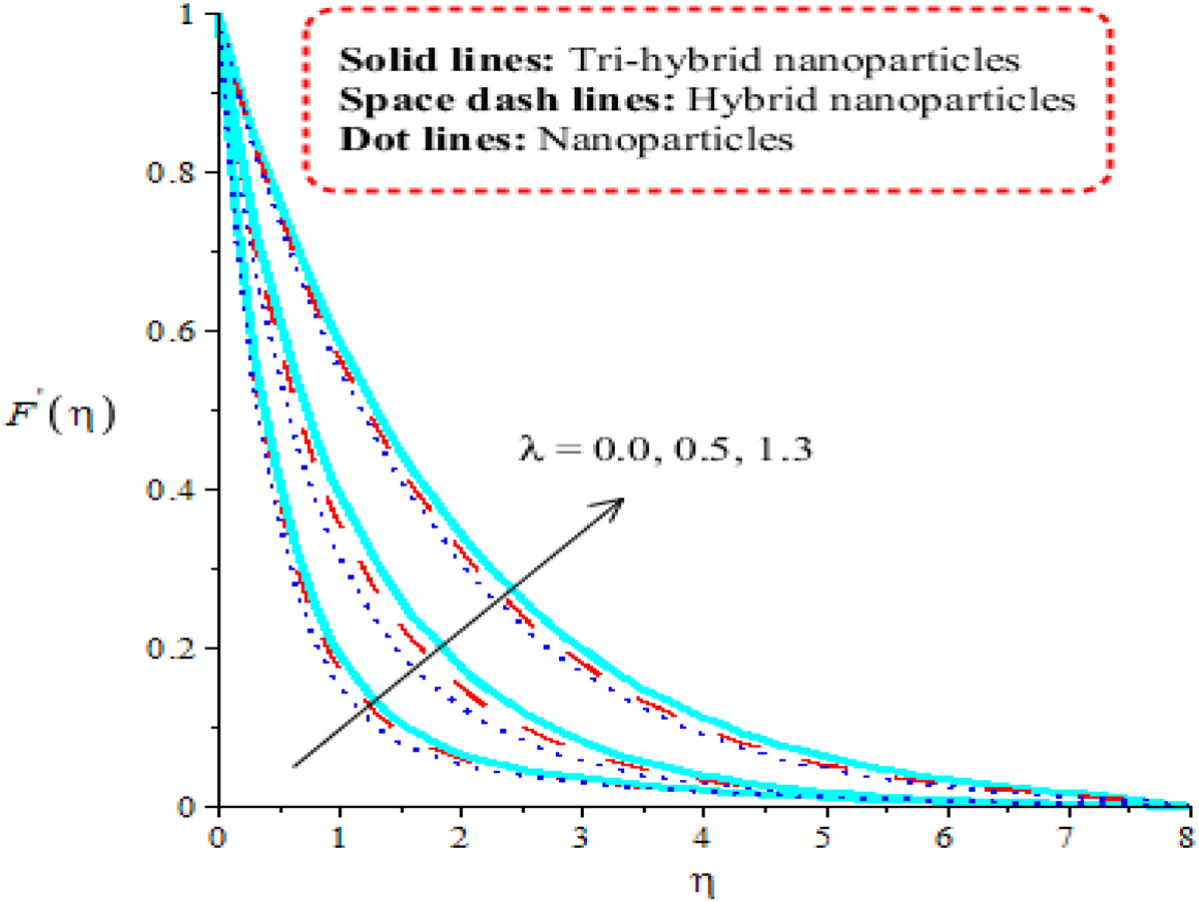
Distribution in ${F}^{^{\prime}}(\eta )$ against $\epsilon .$

Aspects of power law number, porosity number, heat generation number and Forchheimer number on velocity curves are observed. It is depicted that solid curves are made for tri-hybrid nanoparticles and dot curves are designed for impact of nanoparticles whereas space dash curves are constructed for hybrid nanoparticles. Figures 3, 4, 5 and 6 are prepared to capture impacts of nanoparticles, tri-hybrid nanoparticles and hybrid nanoparticles. Figure 3 is made for graphical study among tri-hybrid nanoparticles, nanoparticles and hybrid nanostructures versus distribution in $\epsilon .$ Appearance of $\epsilon$ is made in current model due to impact of porous medium at surface. It is noticed that variation in $\epsilon$ brings declination into motion of fluid particles. Physically, frictional force is produced at surface of sheet against flow of particles. So this frictional force is responsible to produce frictional into particles. Moreover, layers are made by tri-hybrid nanoparticles is more significant rather than layers at boundary are made by nanoparticles and hybrid nanostructures. Physically, fluid particles get fewer spaces among fluid particles. Therefore, flow is slow down. It is observed that Darcian force is directly proportional to permeability regarding medium. Hence, flow becomes decelerate. Figure 4 is tabulated influence of heat generation number versus velocity curves including tri-hybrid nanoparticles. Dual behaviour of heat generation number is addressed against velocity profile whereas phenomena of heat generation (for positive values) and heat absorption (for negative values) are known as dual behaviours. Fluid absorbs more heat energy and fluid runs fast over melting surface. In this approach, tri-hybrid nanoparticles make less thickness into momentum boundary as compared nanoparticles and hybrid nanostructures. Fluid becomes thin using higher numerical values of heat source parameter. Physically, an external heat source is implemented at surface of wall. An external heat source reduces frictional force among fluid layers. Hence, flow becomes accelerate applying argument numerical values of heat source parameter. Figure 5 demonstrates behaviour of power law number on velocity. Appearance of power law number is formulated because of occurrence of pseudo-plastic liquid. This kind of fluid treats as shear thinning and non-Newtonian liquid for $m<1.$ Viscosity of liquid is decreased when power law number is increased. Further, thickness related to momentum boundary is adjusted within variation in power law number. It is observed that flow among layers is slow down when $m$ is increased. It means that viscosity of fluid is decreased applying higher impact of power law parameter. An induced flow by tri-hybrid nanoparticles is enhanced rather than flow is induced by nanoparticles and hybrid nanostructures. Relationship among thermal convective and velocity profile is addressed by Fig. 6. buoyancy forces play a vital role on flow of fluid particles while thermal convective number has relation within temperature difference. Flow is enhanced when $\lambda$ is inclined and speed of flow is increased due to impact of buoyancy forces. MBLT (momentum boundary layer thickness) for ternary hybrid nanoparticles is greater than thickness of momentum boundary layers of hybrid nanoparticles and nanostructures. It is dimensionless parameter which enhances thickness regarding momentum boundary layers. Physically, gravitational acceleration is generated among fluid layer due to appetence $\lambda .$ This gravitational acceleration creates motion into fluid particles.

### Aspects of physical parameters on temperature curves

**Figure 7:**
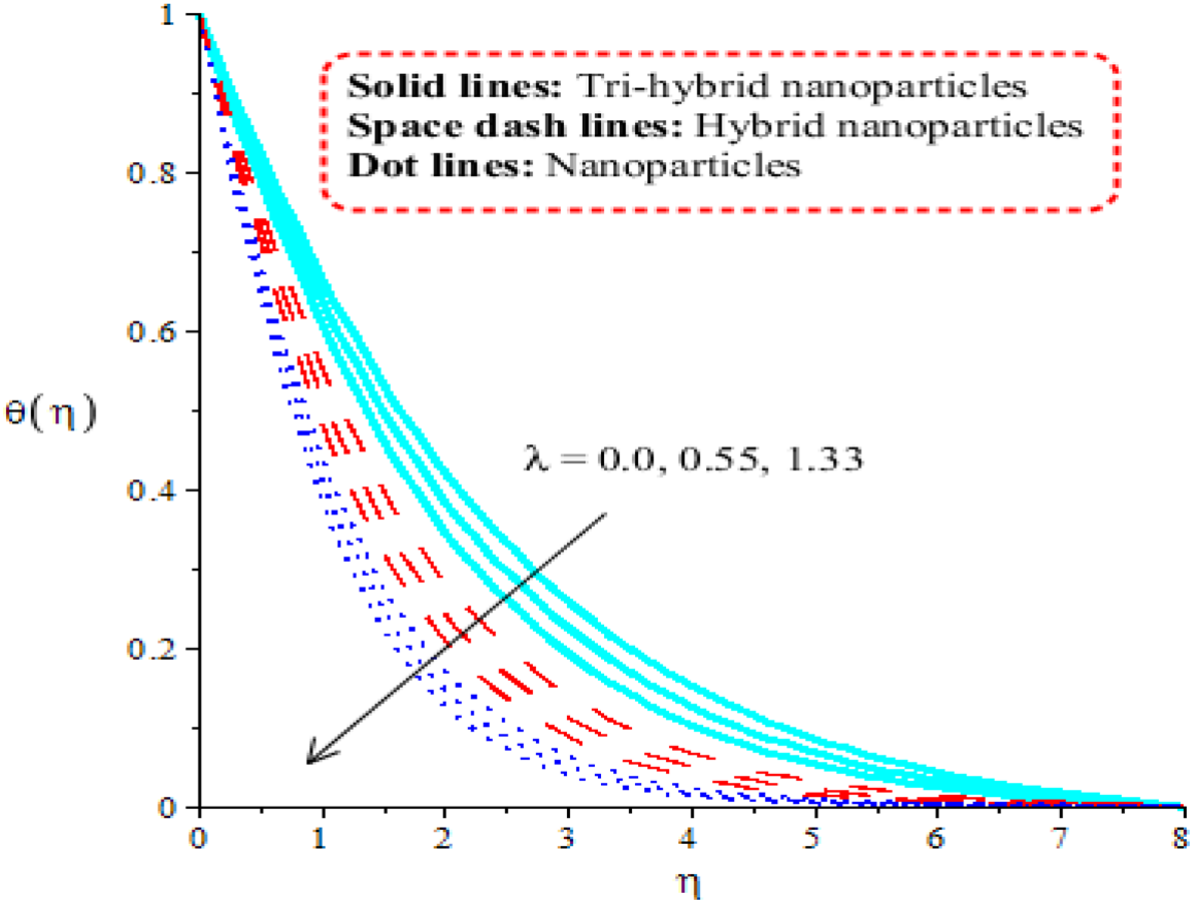
Distribution in $\theta (\eta )$ against $\lambda .$

**Figure 8:**
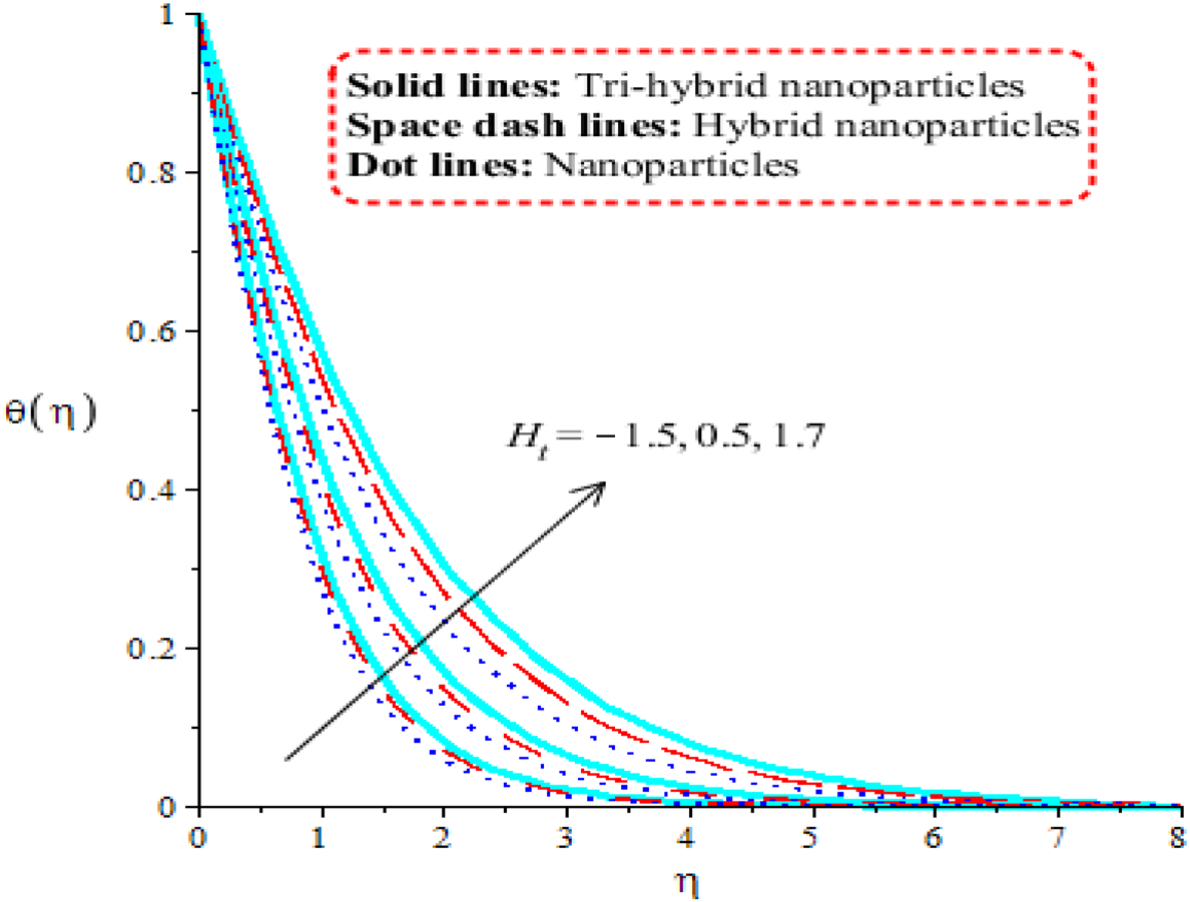
Distribution in ${F}^{^{\prime}}(\eta )$ against ${H}_{t}.$

**Figure 9:**
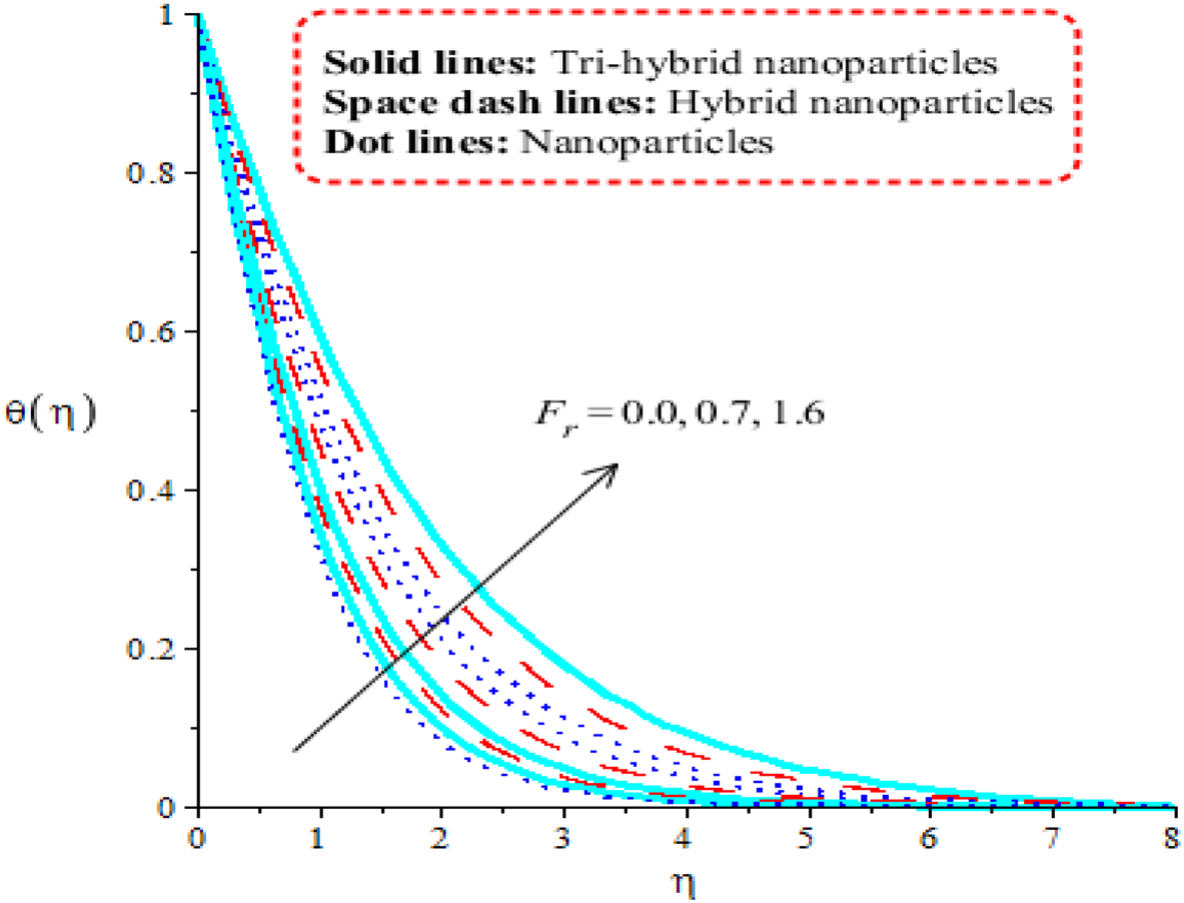
Distribution in $\theta (\eta )$ against ${F}_{r}.$

**Figure 10:**
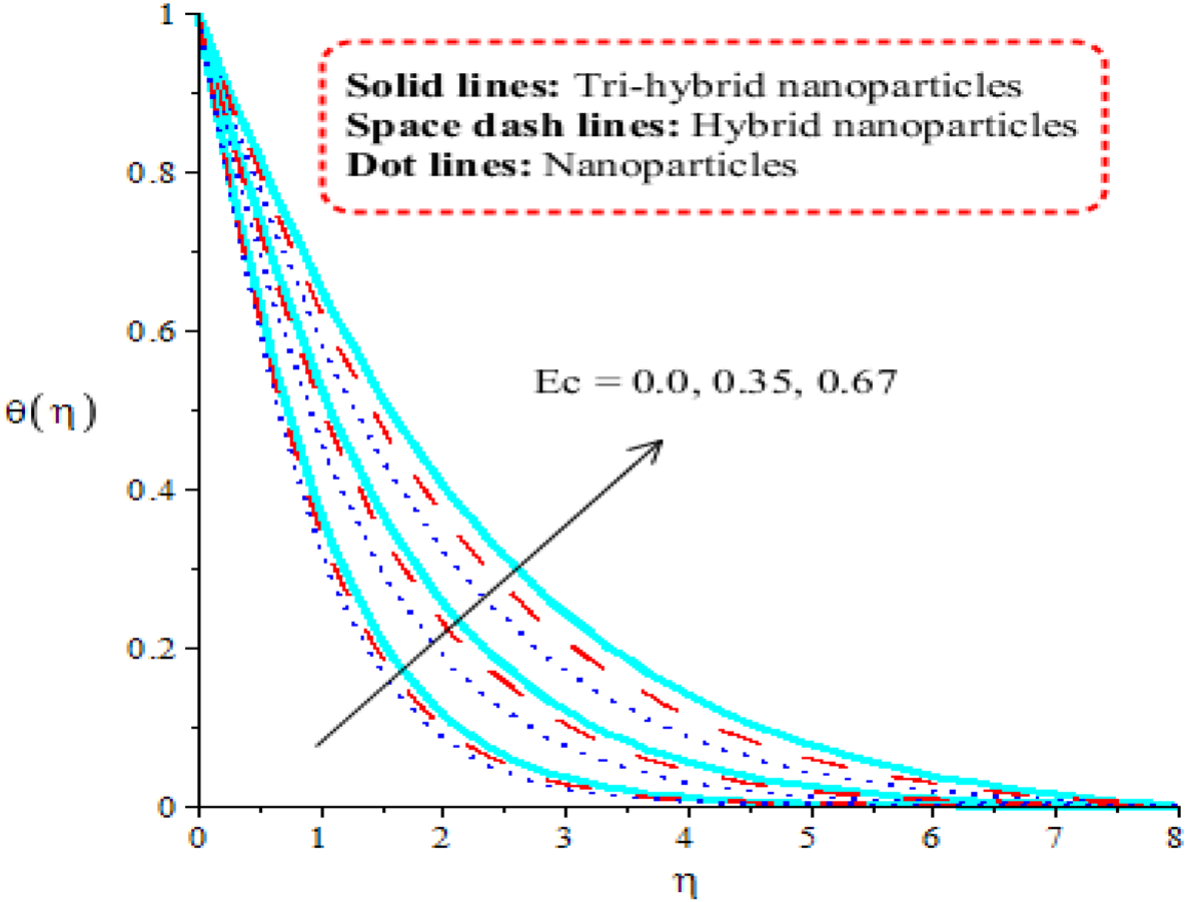
Distribution in $\theta (\eta )$ against ${E}_{c}.$

Comparison among nanoparticles, hybrid nanostructures and tri-hybrid nanostructures on temperature profile is addressed by Figs. 7, 8, 9 and 10 along variation in physical parameters. Figure 7 addresses role of thermal convective number against temperature profile. Buoyancy force is made reason for development of $\lambda$ in current model. Production of thermal energy is reduced with respect to $\lambda$. It is investigated that appearance of $\lambda$ is produced using vertical heated surface. The gravitational acceleration is produced while motion into fluid particles is increased. The fluid particles absorb less heat energy due to gravitational acceleration. Moreover, more production of heat energy is developed by tri-hybrid nanoparticles as compared production of heat energy for nanoparticles and hybrid nanostructures. Figure 8 tabulates heat mechanism versus distribution in heat generation number. Two cases related to heat absorption and heat generation are noticed by Fig. 8. One case is based on heat generation (for positive values) and other case is known as heat absorption (for negative values). Both cases have vital impact on temperature profile. It is demonstrated that temperature of particles is boosted because of external heat source. Moreover, fluid particles absorb more heat energy when external heat source is implemented. Thickness regarding thermal boundary layers is declined using external heat source. Therefore, heat energy can be managed using an impact of heat source parameter. Thermal energy for ternary hybrid nanoparticles is enhanced rather than nanoparticles and hybrid nanomaterial. Figure 9 performs the thermal energy performance against distribution in Forchheimer number. Profile related to thermal energy is increased with respect to distribution in ${F}_{r}$. Impact of ${E}_{c}$ on temperature is noticed by Fig. 10. Existence of $Ec$ is modeled because of viscous dissipation (called work done). More heat energy into particles is absorbed when $Ec$ is increased. Mathematically, $Ec$ appears in viscous dissipation term (in dimensionless energy equation). So, direct relation is found versus heat energy against $Ec$. An impact of Eckert parameter is produced due to the inspiration of viscous dissipation. It is visualized that direct relation among viscous dissipation and heat energy. Therefore, heat energy is inclined sufficiently when $Ec$ is increased. In comparative point of view, efficiency of heat energy is increased for tri-hybrid nanoparticles rather than heat energy is produced by nanoparticles and hybrid nanostructures.

### Aspects of velocity and temperature gradients

Variation aspects of velocity gradient and temperature gradient are measured against change in heat generation/absorption and thermal convective, Forchheimer numbers. Table 4 depicts aspects of velocity and temperature gradients considering tri-hybrid nanoparticles. Vital role of heat generation and heat absorption on velocity and temperature gradient is observed. Reduction in temperature gradient and velocity gradient is captured versus positive as well as negative values of heat generation number. Opposite impact is noticed on velocity gradient and temperature gradient versus change in thermal convective number. Temperature gradient is enhanced with respect to large values of heat generation number. Forchheimer number boosts impact of temperature gradient but velocity gradient is reduced when ${F}_{r}$ is increased. Table 5 predicts influence regarding rate of heat transfer and flow rate against argument numerical values of $Ec, Pr$ and $\epsilon .$ It is estimated that heat transfer rate is declined versus higher numerical values $Ec$ but declination into flow rate is noticed against an impact of $Ec.$ The rate of heat transfer is boosted when $Pr$ is increased. Skin friction coefficient is enhanced by applying higher numerical values $\epsilon .$

**Table 4:** Variation in skin friction coefficient and temperature gradient versus distribution in ${H}_{t}$, ${F}_{r}$ and $\lambda$ including tri-hybrid nanoparticles.

		$-{{\left(Re\right)}^{\frac{1}{m+1}}C}_{f}$	$-{\left(Re\right)}^{\frac{-1}{m+1}}Nu$
${H}_{t}$	− 1.5	0.42797718	0.55852906
	0.1	0.41870728	0.76092060
	0.7	0.40781438	0.64303994
	0.0	0.20881888	1.5281956
${F}_{r}$	0.5	0.36868534	1.3571423
	0.7	1.3571423	1.3038192
	0.3	0.35320488	1.3720856
$\lambda$	0.6	0.15365893	1.3894693
	0.8	0.029884079	1.4646254

**Table 5:** Thermal aspects of Nusselt number and skin friction coefficient versus $Ec, Pr$ and $\epsilon .$

Variation in parameters		${-{\left(Re\right)}^{\frac{1}{m+1}}C}_{f}$	${-\left(Re\right)}^{\frac{-1}{m+1}}Nu$
$Ec$	0.0	0.81715589	1.3564572
	0.3	0.82745549	1.2935174
	0.7	0.81653926	1.1204841
	0.0	0.84463158	1.6390042
$Pr$	206	0.83190583	1.7503621
	208	0.81849360	1.8555670
	0.0	1.0182082	1.7389999
$\epsilon$	0.6	1.8221094	1.8817948
	1.2	1.9434104	1.9379620

## Key findings

Rheology of pseudo-plastic liquid is injected into thermal energy along with Darcy’s Forchheimer theory towards a heated plate. Buoyancy forces, heat generation, tri-hybrid nanoparticles and viscous dissipations are considered. Velocity and temperature gradients are addressed. A strong scheme called finite element approach is adopted to compute results of current analysis. Key points of current investigation are discussed below.

- 330 elements are needed for convergence of current analysis.
- Motion and temperature into fluid particles are boosted versus enhancement in heat generation number.
- Ternary hybrid nanostructures played significant impact to enhance thermal energy and motion of fluid particles rather than nanoparticles and hybrid nanostructures particles.
- Argumentation into thermal boundary layers are inclined versus change in Forchheimer and Eckert number.
- Temperature field grows against Eckert number.
- Forchheimer and Darcy numbers bring slowness into flow of particles.

## Data Availability

The datasets generated/produced during and/or analyzed during the current study/research are available from the corresponding author on reasonable request.

## List of symbols

$$y,x$$: Space coordinates
$$\nu$$: Kinematic viscosity
FEM: Finite element method
$m$: Power law number
${k}^{s}$: Permeability within porous medium
$$\rho$$: Fluid density
$${T}_{\infty }$$: Ambient fluid
$$bf$$: Base fluid
$$\theta$$: Dimensionless temperature
$$\mu$$: Dynamic viscosity
${U}_{w}$: Wall velocity along horizontal direction
$$\infty$$: Infinity
$$\Psi$$: Stream function
$$\epsilon$$: Porosity parameter
$$\lambda$$: Ratio velocity
$$Ec$$: Eckert number
$${\varphi }_{1}, {\varphi }_{3}, {\varphi }_{2}$$: Volume fractions
$${\tau }_{w}$$: Wall temperature
${C}_{f}$: Skin friction coefficient
$${w}_{3}, {w}_{2}, {w}_{1}$$: Weight functions
$$U$$: Composite velocity
$${u}_{1}, {u}_{2}$$: Velocity components
$Thnf$: Tri-hybrid nanoparticles
$\gamma$: Coefficient of thermal expansion
${F}_{D}$: Inertia coefficient in term of porous medium
$$G$$: Gravitational acceleration
$$T$$: Temperature
$${T}_{w}$$: Wall temperature
$$f$$: Fluid
$$F$$: Dimensionless velocity
${C}_{p}$: Specific heat capacity
${V}_{w}$: Wall velocity along vertical direction
$$\eta$$: Independent variable
PDEs: Partial differential equations
$${F}_{R}$$: Forchheimer parameter
$$Pr$$: Prandtl number
${H}_{t}$: Heat source number
$\sigma$: Fluid electric conductivity
$$Re$$: Reynolds number
$$Nu$$: Nusselt number
${Q}_{w}$: Heat flux at wall
$$\psi$$: Shape function
