# Author Correction: Finite element analysis for ternary hybrid nanoparticles on thermal enhancement in pseudo-plastic liquid through porous stretching sheet

**DOI:** 10.1038/s41598-022-20206-7

**Published:** 2022-09-13

**Authors:** Muhammad Sohail, Essam R. El-Zahar, Abd Allah A. Mousa, Umar Nazir, Saad Althobaiti, Ali Althobaiti, Nehad Ali Shah, Jae Dong Chung

**Affiliations:** 1 Department of Mathematics, Khwaja Fareed University of Engineering & Information Technology, Rahim Yar Khan, 64200 Pakistan; 2 Department of Mathematics, College of Science and Humanities in Al-Kharj, Prince Sattam Bin Abdulaziz University, P.O. Box 83, Al-Kharj, 11942 Saudi Arabia; 3 Department of Basic Engineering Science, Faculty of Engineering, Menoufia University, Shebin El-Kom, 32511 Egypt; 4 Department of Mathematics, College of Science, Taif University, P.O. Box 11099, Taif, 21944 Saudi Arabia; 5 Department of Applied Mathematics and Statistics, Institute of Space Technology, P.O. Box 2750, Islamabad, 44000 Pakistan; 6 Department of Sciences and Technology, Ranyah University Collage, Taif University, P.O. Box 11099, Taif, 21944 Saudi Arabia; 7 Department of Mechanical Engineering, Sejong University, Seoul, 05006 Korea

Correction to: *Scientific Reports* 10.1038/s41598-022-12857-3, published online 02 June 2022

In the original version of this Article Muhammad Sohail was incorrectly affiliated with ‘Department of Mechanical Engineering, Sejong University, Seoul, 05006, Korea.’ The correct affiliation is listed below.

Department of Mathematics, Khwaja Fareed University of Engineering & Information Technology, Rahim Yar Khan, 64200, Pakistan.

The original Article has been corrected.

